# Total Intestinal Atresia in a Neonate: A Case Report

**DOI:** 10.7759/cureus.82082

**Published:** 2025-04-11

**Authors:** Cory Nonnemacher, Seth Saylors, Naomi-Liza Denning, Vatche Melkonian, Pablo Aguayo

**Affiliations:** 1 Pediatric Surgery, Children's Mercy Hospital, Kansas City, USA

**Keywords:** bilious emesis, intestinal atresia, neonatal obstruction, neonatology, total intestinal atresia

## Abstract

Intestinal atresias are a common cause of neonatal intestinal obstruction. An extremely rare variant is total intestinal atresia, where no lumen is appreciable from the duodenum through the colon. We describe a case of a 33-week female who was born at an outside hospital and underwent laparotomy for concern for intrauterine perforation and proximal intestinal obstruction. Laparotomy revealed a duodenal perforation and an atresia in the proximal jejunum. Resection of this segment revealed no intestinal lumen distally, and the patient was left in discontinuity and transferred to our tertiary children’s hospital. Repeat laparotomy revealed atretic bowel with no lumen through the colon with a normal appearing mesentery. A decompressive gastrostomy tube was placed, and the patient passed away after withdrawal of care. Total intestinal atresia is a rare variation of intestinal atresia with no current surgical or medical treatments.

## Introduction

Intestinal atresias are a common cause of neonatal intestinal obstruction, occurring in one in 5,000 to one in 14,000 live births [1]. Jejunal and ileal atresia, often referred to as jejunoileal atresia (JIA), can be diagnosed prenatally or most commonly postnatally with signs of intestinal obstruction such as emesis, proximally distended bowel, air fluid levels, and absence of distal bowel gas [1]. The pathogenesis of JIA is believed to be intrauterine vascular accidents during late gestation, leading to resorption of the necrotic fetal bowel [2-4]. This pathogenesis differs from duodenal atresia, which is believed to be caused by a failure of recanalization of the bowel lumen [5]. Finally, colonic atresia is a rare entity occurring in around one in 66,000 live births and tends to be associated with other congenital defects such as abdominal wall defects, JIAs, and Hirschsprung disease [6,7]. Total intestinal atresia is a rare entity with few reports of it in the literature. Morris-Stiff et al. in 1998 reported a case with prenatally diagnosed gastroschisis; however, at birth, the abdominal wall defect was closed and on laparotomy, total-intestinal atresia was noted tracking from the jejunum up to the umbilicus, and the remainder of the atretic bowel tracking down from the umbilicus to the rectum [8]. Aggerwal et al. reported a case of a normal-term child born with intestinal obstruction that was found on exploration to be an intestinal atresia with a singular small defect in the intestinal mesentery, with the remainder of the bowel being atretic with normal-appearing mesentery [9]. While not mentioned in other reported cases, severe combined immunodeficiency (SCID), a primary immune deficiency with dysfunction of both T and B cell lineages, has been associated with intestinal failure. Fullerton et al. described high rates of SCID screening in infants with intestinal failure, including patients with necrotizing enterocolitis, gastroschisis, motility disorders, and intestinal atresia [10]. Mutations in the TTC7A gene are the cause of both familial multiple intestinal atresias and SCID and are likely are related to intestinal atresias and potentially total intestinal atresia [11]. This finding led the authors to question if the etiology of total intestinal atresia is a vascular accident or more likely a failure of recanalization of the bowel lumen, more similar to duodenal atresia. We report a case of a 33-week baby delivered with an initial diagnosis of intrauterine bowel perforation who, on exploration, was found to have a duodenal perforation and an associated total intestinal atresia.

## Case presentation

A 33-week-3-day-old female neonate was born at an outside hospital and admitted to their neonatal intensive care unit (NICU). The mother was a 38-year-old G10P9 who had no prior medical history and received limited prenatal care, but denied any intrapartum substance abuse. Initial evaluation at the outside hospital revealed no physical abnormalities, and post-partum echocardiogram showed a 1.1 mm ventral septal defect and small patent ductus arteriosus with bidirectional flow. Abdominal imaging showed concerns for a proximal obstruction, and the patient was taken to the operating room at that facility, where a duodenal perforation was repaired primarily and an atretic segment of proximal jejunum just distal to the duodenum was encountered. This segment was absent from the mesentery. A bowel resection of the atretic segment was performed, but no lumen was encountered distally after multiple short resections of distal bowel, so the patient was left in discontinuity and closed. After stabilization, the patient was transferred to our tertiary children’s hospital for definitive care.

**Figure 1:**
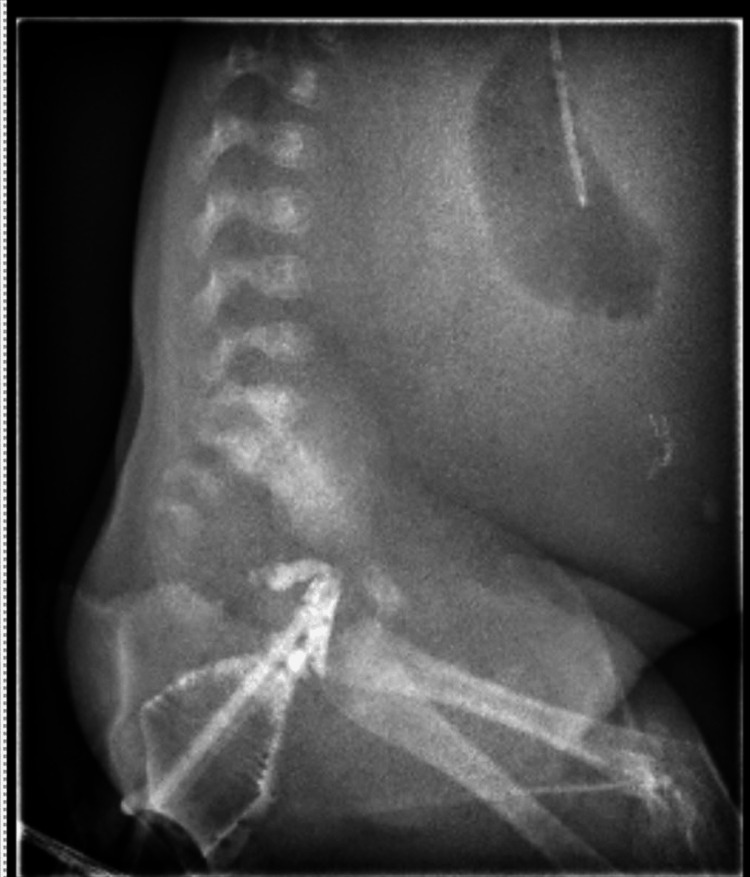
Contrast enema showing no passage of contrast proximally in the rectum

On arrival, the patient was stable. An orogastric tube (OGT) was in place with bilious drainage, and the anus had a normal appearance and location. A decision was made to proceed with imaging workup with a contrast enema to delineate anatomy. Figure 1 shows a contrast enema with approximately 2.3 cm of distal colon filling with no proximal filling and no bowel gas outside of the gastric bubble.

Extensive discussion occurred with family regarding the concern for an extensive intestinal atresia versus a total intestinal atresia, which would likely be nonsurvivable. The family elected to proceed forward with exploration for definitive diagnosis.

The patient was brought to the operating room for exploration. Extensive lysis of adhesions was required for exposure of the underlying bowel. There was a normally appearing but dilated stomach, as well as duodenum to the prior bowel resection location. The remainder of the bowel was grossly normal appearing, giving off the appearance of diverted small bowel. The mesentery was formed and intact with a normal appearance. There was 51 cm of small bowel remaining at this point. Enterotomies were made at 5, 20, 30, and 40 cm of the small bowel. At all sites, the lumen was obliterated and contained a white cord-like tissue centrally. A colotomy was also made in the ascending colon and had no lumen, with similar white cord-like tissue. A decision was made to place a gastrostomy tube for venting purposes and close.

The patient was managed in the NICU for eight days on total parenteral nutrition (TPN) and gastric decompression, at which point the family decided to compassionately withdraw care and the patient went home with family, passing away three days later. Of note, the patient’s newborn screening returned positive for severe combined immunodeficiency (SCID).

## Discussion

Total intestinal atresia is a rare variant of intestinal atresia, with few reports of it in scientific literature. All reported cases that the authors identified were fatal, with compassionate withdrawal of care performed in each instance [8,9]. Recognition of total intestinal atresia has been postnatal in all reports, with early diagnosis of proximal intestinal obstruction. While additional imaging could be performed, no test will be definitive outside of performing surgical exploration. In our case, the patient underwent exploration at an outside facility, where the first stages of the diagnosis were being established. It was reported that a proximal segment of the jejunum was absent of its mesentery and atretic, but resection revealed no distal lumen, so efforts were halted at that point in an attempt to potentially avoid iatrogenic short gut. We performed a contrast enema on arrival at our facility to give us information about the distal bowel, which revealed a distal obstruction in conjunction with our known proximal obstruction. While this could have potentially represented an isolated colonic atresia in conjunction with a JIA, our suspicion at this point was for total intestinal atresia. Operative exploration at our facility revealed normal mesentery, but the bowel from the ligament of Treitz distally had no appreciable lumen, and decompressive gastrostomy was performed.

The patient was already in discontinuity and therefore a contrasted upper GI study would not have revealed any new information prior to exploration. The expectation for an upper GI study would be no passage of contrast distal to duodenum. The contrast enema in our case is as you would expect for total intestinal atresia.

Grosfeld et al. described the classification and management of JIA in 1979 [12]. They described type 1 to have an internal membrane with serosal continuity and no mesenteric defect; type 2 involved proximal and distal blind pouches connected by a fibrous cord; type 3a involved a V-shaped mesenteric defect without a fibrous cord and type 3b having a coiled “apple peel” deformity; and finally type 4 being multiple atretic areas [12].

The patient screened positive for SCID, a primary immune deficiency with dysfunction of both T and B cell lineages. Given this correlation previously mentioned, it is likely related in our case but was not explored further while the patient was alive, as this resulted shortly after the patient passed. As sepsis is the leading cause of death in patients with intestinal failure, it warrants further exploration for patients who survive past the initial operative period.

It is difficult, if not impossible, to establish the pathogenesis of this type of atresia based on a few isolated reports. In our patient, as well as the one Aggerwal et al. [9] described, the patient had a single type 3a atresia with the remainder of the distal bowel being atretic with mesentery having a normal appearance [9]. While they hypothesize this to be secondary to a failure of recanalization, this does not align with the accepted embryology. During the fourth to eighth week of gestation, rapid epithelial growth in the foregut obliterates the lumen, which recanalizes beginning in the eighth to tenth weeks [13]. The midgut and the hindgut, in contrast, do not lose their lumen and remain in communication with the fetal yolk sac via the omphalomesenteric duct [14]. It is unclear in cases of total intestinal atresia if intrauterine vascular accidents are the etiology, as is the case in an isolated atresia.

This case report is limited in that we did not send any specimen for pathology and pathologic analysis may shine light onto the pathophysiology of this type of atresia. This is also limited in being a single patient case report, but given the nature of this rare disease, it is unlikely a single provider would encounter this more than once in their career.

## Conclusions

Total intestinal atresia is an extremely rare variation of congenital intestinal atresia. Limited reports exist with all patients passing with compassionate withdrawal of care. Our case highlights a neonate born with findings consistent with bowel obstruction and operative intervention, and imaging findings confirmed the diagnosis. While contrast enema and potentially upper gastrointestinal contrasted imaging may help with the workup of a patient, only intra-operative findings showing no lumen through the bowel are diagnostic. Treatment after diagnosis focuses on palliation with no current surgical or medical cure existing.

## References

[1] Adams SD, Stanton MP (2014). Malrotation and intestinal atresias. Early Hum Dev.

[2] Frischer JS, Azizkhan RG (2012). Jejunoileal atresia and stenosis. Pediatric Surgery, 7th Edition.

[3] Louw JH (1959). Congenital intestinal atresia and stenosis in the newborn. Observations on its pathogenesis and treatment. Ann R Coll Surg Engl.

[4] Puri P, Fujimoto T (1988). New observations on the pathogenesis of multiple intestinal atresias. J Pediatr Surg.

[5] Prasad TR, Bajpai M (2000). Intestinal atresia. Indian J Pediatr.

[6] Davenport M, Bianchi A, Doig CM, Gough DC (1990). Colonic atresia: current results of treatment. J R Coll Surg Edinb.

[7] Etensel B, Temir G, Karkiner A, Melek M, Edirne Y, Karaca I, Mir E (2005). Atresia of the colon. J Pediatr Surg.

[8] Morris-Stiff G, al-Wafi A, Lari J (1998). Gastroschisis and total intestinal atresia. Eur J Pediatr Surg.

[9] Aggerwal N, Sugandhi N, Kour H, Chakraborty G, Acharya SK, Jadhav A, Bagga D (2019). Total intestinal atresia: revisiting the pathogenesis of congenital atresias. J Indian Assoc Pediatr Surg.

[10] Fullerton BS, Velazco CS, Hong CR, Carey AN, Jaksic T (2018). High rates of positive severe combined immunodeficiency screening among newborns with severe intestinal failure. JPEN J Parenter Enteral Nutr.

[11] Yang W, Lee PP, Thong MK (2015). Compound heterozygous mutations in TTC7A cause familial multiple intestinal atresias and severe combined immunodeficiency. Clin Genet.

[12] Grosfeld JL, Ballantine TV, Shoemaker R (1979). Operative mangement of intestinal atresia and stenosis based on pathologic findings. J Pediatr Surg.

[13] Wells G (2013). The small intestine. Diagnostic Imaging of Infants and Children.

[14] Malone JC, Arbor TC, Shah AB (2023). Embryology, midgut. StatPearls.

